# Good Dietary Control Significantly Improves Anthropometric and Metabolic Parameters and Liver Function in Patients with Type 2 Diabetes Mellitus—A Pilot Study

**DOI:** 10.3390/nu18020222

**Published:** 2026-01-10

**Authors:** Bogusława Luzak, Patrycja Szymańska, Marcin Kosmalski

**Affiliations:** 1 Department of Hemostasis and Hemostatic Disorders, Medical University of Lodz, Mazowiecka 6/8, 92-215 Lodz, Poland; boguslawa.luzak@umed.lodz.pl (B.L.); patrycjaszymanska@op.pl (P.S.); 2 Faculty of Economics, WSB Merito University in Opole, Augustyna Kosnego 72, 45-372 Opole, Poland; 3 Department of Clinical Pharmacology, Medical University of Lodz, Kopcinskiego 22, 90-153 Lodz, Poland

**Keywords:** type 2 diabetes mellitus, nutrition in diabetes care, fiber-enriched diet, diet adherence, liver function

## Abstract

**Background/Objectives**: The aim of the study was to analyze dietary and lifestyle adherence in patients with type 2 diabetes mellitus (T2DM) under the care of a diabetes clinic. **Methods**: The study included two groups: patients under the close control of a dietitian (n = 50) who followed a standard (DD) or fiber-enriched diabetic diet (FD), and patients under the care of a diabetes clinic without close supervision of a dietitian (n = 50). **Results**: After 3 months, both DD and FD significantly improved metabolic control in the patients under the care of a dietitian. However, FD was slightly better compared to DD (BMI reduction by an average of 2.4% (95% CI: 1.6%; 3.1%) for DD vs. 4.8% (95% CI: 3.6%; 6.0%) for FD; waist circumference decreasing 2.0% (95% CI: 1.2%; 3.4%) for DD vs. 3.5% (95% CI: 2.6%; 4.3%) for FD, *p* < 0.01; glycemia reduction 19.9% (95% CI: 14.8%; 25.0%) for FD vs. 5.6% (95% CI: 1.9%; 9.3%) for DD, *p* < 0.001; GGTP activity reduction 35.7% (95% CI: 28.9%; 42.5%) for FD vs. 1.8% (95% CI: −15.2%; 18.3%) for DD, *p* < 0.001). In addition, only half of the patients without the close supervision of a dietitian declared adherence to the diet. Most respondents had a satisfactory level of nutritional knowledge, but the analysis indicates the weakly marked influence of the protective features of nutrition as well as evidence of the low contribution of an unhealthy diet. **Conclusions**: Considering the level of nutritional knowledge and low awareness of their health condition in many patients, visits to the doctor and brief nutrition education are not enough for dietary care in T2DM patients. A dietitian’s care is necessary to improve their health.

## 1. Introduction

Type 2 diabetes mellitus (T2DM) is the most common form of diabetes, accounting for over 90% of all cases worldwide [1]. Diabetes induces susceptibility to a number of serious microvascular (retinopathy, neuropathy, and diabetic kidney disease) and macrovascular complications (coronary artery diseases and ischemic and hemorrhagic stroke) that drastically affect the patient’s quality of life [2]. Additionally, studies have demonstrated links between diabetes and a broad range of comorbidities such as cancers, mental disorders (depression, anxiety, eating disorders, sleep disorders, cognitive disability), susceptibility to infections, and excessive fatty liver infiltration and its consequences [3,4]. The risk of these consequences can be significantly reduced by implementing appropriate therapeutic procedures aimed not only at proper metabolic control of the disease but also at its pleiotropic properties.

Effective treatment of T2DM is multifaceted and requires a combination of pharmacological treatment, lifestyle modifications, and particularly physical activity and dietary interventions. Currently, there is a strong emphasis on whole foods and dietary patterns. Nutrient-dense foods such as vegetables, fruits, and legumes are recommended and healthy eating habits are encouraged to improve overall health, help achieve weight and glycemic goals, and prevent complications. There is increasing appreciation of the critical role that diet plays not only in controlling blood glucose levels but also in influencing a wide range of metabolic and hepatic parameters. Dietary strategies to improve insulin sensitivity, promote weight loss, and reduce cardiovascular risk factors are crucial to comprehensive diabetes care [5].

Recently, despite changes in the recommended dietary approach for people with T2DM, appropriate dietary patterns are still promoted, including macronutrient approaches (low-glycemic-index diet, high-fiber diet, high-monounsaturated-fatty-acid diet, low-carbohydrate diet, high-protein diet), Mediterranean diets, alternative diets (e.g., vegetarian, Dietary Approaches to Stopping Hypertension diet, Portfolio diet, Nordic diet, Atkins diet), dietary patterns including specific foods (legumes, fruits and vegetables, nuts, whole grains, dairy) and meal replacement, as defined by guidelines [5]. Among these strategies, increased dietary fiber intake has attracted considerable attention due to its multifaceted benefits demonstrated in recent clinical and experimental studies. These have shown that increasing fiber intake can reduce the risk of various chronic diseases, such as cardiovascular disease, T2DM, obesity, colon cancer, and inflammation [6].

The choice of one dietary pattern is sometimes very difficult and depends on the decision of the patient and the dietitian/doctor. Above all, a significant problem is resisting the temptation of tasty food. In addition, it is worth emphasizing the influence of social factors (including parenting style and household eating habits), product-related factors (including product attributes, packaging, and labels), personal factors (e.g., food knowledge and culinary skills) and situational factors (food availability, time pressure, and store design) [7]. In addition, the presence of excess body weight, cardiovascular diseases, metabolic disorders, cancer, and kidney, liver, and gastrointestinal dysfunctions also have a significant impact on the choice of a specific diet [8]. So far, a wide range of diets have been proposed, including low-carbohydrate, low-fat, high-fat, and high-protein diets, detoxification diets, and others of Mediterranean or Paleolithic origin. Most of these diets are used to reduce body weight, normalize glycemia, and improve liver function. It should be emphasized that these diets have very diverse support for their effectiveness and safety in scientific evidence [9]. Therefore, the clinical question arises, what has the greatest impact on the effectiveness of a diet—its type or the use of appropriate dietary patterns?

According to the Standards of Care in Diabetes recommended by the Polish Diabetes Society, the goal of dietary treatment for individuals with diabetes is to maintain normoglycemia, optimal blood pressure values and serum lipid and lipoprotein levels, and appropriate body weight, and these targets can be achieved using individually calculated caloric intake, distribution of calories among daily meals, selection of food sources providing necessary energy, vitamins, minerals, and phytochemicals, and moderation of products whose consumption should be limited [10]. Generally, the dietary education of patients on the general principles of proper nutrition in diabetes is provided at diagnosis by authorized persons (a doctor, dietitian, or diabetic nurse) and is verified during visits to the diabetes clinic. However, the adherence of T2DM patients to the recommendations for treatment, diet, and lifestyle is highly variable and often inadequate [11,12,13].

Therefore, the aim of our study was to analyze the adherence to a diabetic diet in T2DM patients remaining under the care of a diabetes clinic. The study included two groups: (1) patients under the care of a diabetes clinic and the close control of a dietitian who followed a standard or fiber-enriched diabetic diet and (2) patients under the care of a diabetes clinic, however, without close supervision of a dietitian. Our research hypothesis assumes that patients who follow a diet excluding monosaccharides and added sugars and increasing dietary fiber intake, and who are under the regular care of a dietitian, achieve improvements in anthropometric and biochemical parameters. In contrast, many patients attending a diabetes clinic without dietitian support do not follow any specific diet and do not pay attention to their lifestyle, despite their doctor’s recommendations. In this study, we introduce the concept of “good dietary control”, which refers to consistent adherence to individually tailored dietary recommendations under the regular supervision of a dietitian. This includes appropriate energy intake, limitation of simple sugars, increased dietary fiber consumption, and continuous monitoring with dietary adjustments. We assume that the obtained results will indicate the need for further research in this area, which may contribute to improving the care of patients with diabetes in Poland and emphasize the need to improve patients’ access to dietitians.

## 2. Materials and Methods

### 2.1. Study Participants

The participants in the study were T2DM patients at the diabetes clinic at the Norbert Barlicki University Teaching Hospital No. 1 of the Medical University of Lodz, Poland. The experiments were approved by the Ethics of Research in Human Experimentation Committee at the Medical University of Lodz (approval No. RNN/452/18/KE, 10 December 2018), and all participants gave their informed written consent to participate in the study. All participants underwent a comprehensive physical examination, including blood pressure measurements. Anthropometric measurements, such as body weight, height, and waist circumference, were also performed and were used as the basis for calculation of body mass index (BMI), waist-to-hip ratio (WHR), and waist-to-height ratio (WHTR). The patients recruited for the study (n = 100) were divided into two equinumerous groups: (1) those under the care of a diabetes clinic and the close control of a dietitian who followed a standard or fiber-enriched diabetic diet (n = 50) and (2) those under the care of a diabetes clinic but without close supervision of a dietitian (n = 50).

### 2.2. Determination of the Anthropometric and Metabolic Effects of a Standard or Fiber-Enriched Diabetic Diet in T2DM Patients Following the Diet Under the Close Control of a Dietitian

The first group consisted of 50 T2DM patients (76% women and 24% men) aged from 36 to 76 years who were under close control of a dietitian. The characteristics of this group are presented in Table 1. For every participant, data on the duration of diabetes, diabetes complications, and treatment were collected, as well as a blood sample being taken for the determination of lipid profile (total cholesterol, triglycerides, LDL-cholesterol, HDL-cholesterol), aspartate and alanine aminotransferase (AST and ALT) activity, gamma glutamyl transferase (GGTP) activity, fasting glucose and creatinine concentration, and HbA1c fraction. Laboratory parameters were measured using the standard diagnostic procedures. The estimated glomerular filtration rate (eGFR) value was calculated using the CKD-EPI formula according to serum creatinine concentration, age, and sex, using mean creatinine and age. Each patient underwent assessments for the Hepatic Steatosis Index (HSI) and the Fatty Liver Index (FLI), along with an abdominal ultrasound, to evaluate the presence of non-alcoholic fatty liver disease (NAFLD). The comorbidities and medications used by patients recruited to study are presented in Table S1 (Supplementary Material). None of the patients used GLP-1 receptor agonist. There was no significance difference in the distribution of comorbidities and medications used between the groups (FD vs. DD). During the study, there were no changes in the treatment administered to the patients.

After qualification for the study, the patients were randomly assigned to either the group using the standard diabetic diet or the group using the fiber-enriched diabetic diet. Randomization (1:1) was performed by an independent researcher using computer-generated random table numbers, with a varied block size (4–8 allocations per block). The patients were under the close supervision of a dietitian. The standard diabetic diet (DD) included complex carbohydrates with a glycemic index < 50 (50% of daily energy requirements for adults), fats (30% of daily energy requirements for adults), and proteins (20% of daily energy requirements for adults) in the proportion of 1:1 for animal to plant proteins. This diet excluded monosaccharides and added sugars. The second group used a diabetic diet enriched with fiber (FD), which included more food fiber (35 g of fiber per day compared to 25 g/day for DD), especially water-insoluble fractions, monounsaturated fatty acids, and products with reduced cholesterol content. After 3 months on these diets, for every participant, anthropometric measurements (body mass, waist circumference) were collected, and a blood sample was taken for determination of lipid profile, AST, ALT, and GGTP activity, creatinine and fasting glucose concentration, and HbA1c fraction.

The effects of the diets were analyzed by comparison of the results after 3 months vs. the results obtained before diets. Also, the efficacy of the diet was estimated as a percent value based on the results before and after the 3-month diets (100% − (t3/t0 × 100%)).

### 2.3. Analysis of Diet Quality Scores and Nutritional Knowledge in Patients Under the Care of a Diabetes Clinic but Without Close Supervision of a Dietitian

In the second group, there were 50 T2DM patients (48% women and 52% men) aged from 25 to 73 years, remaining under the care of a diabetes clinic but without the close supervision of a dietitian. The participants were divided into two subgroups based on their responses to a question about ongoing dietary adherence—those claiming not to be on a diet (referred to as the Non-Diet group, n = 25) and those claiming to be on a diet (referred to as the Diet group, n = 25), respectively. The basic characteristics of these participants groups are presented in Table 2. The respondents who were qualified for the Diet group pointed most often to a diabetic diet, a carbohydrate-restricted diet, a sugar-restricted diet, a low-glycemic-index diet, and a diet that excluded sweets as the type of diet they followed. A total of 72% (n = 18) of this subgroup followed a diet on a doctor’s recommendation and 28% (n = 7) followed a diet by their own choice.

Diet quality scores (DQS) and nutritional knowledge were assessed using the Dietary Habits and Nutrition Beliefs Questionnaire (KomPAN, The Committee of Human Nutrition, Polish Academy of Science). KomPAN included food frequency consumption of thirty-three food items and was formulated by six diet indexes: pro-Healthy-Diet-Index (pHDI-10), non-Healthy-Diet-Index (nHDI-14), high-Glycemic-Diet-Index-7 (hGIDI-7), low-Glycemic-Diet-Index-4 (lGIDI-4), high-Sugar-Diet-Index-4 (hSDI-4), and high-Saturated-Fats-Diet-Index-8 (hSFDI-8) [14,15].

### 2.4. Statistical Analysis

Statistical analysis was performed in Statistica 13.1 (Statsoft, Cracow, Poland) and GraphPad Prism 8 (San Diego, CA, USA). The normality of distribution of the analyzed variables was assessed using the Shapiro–Wilk test. The data in tables and figures were presented as mean ± standard deviation or as median and interquartile range: Me (Q1; Q3). For normally distributed variables, the statistical significance of differences between the two groups was estimated using a paired Student’s *t*-test (comparison between after the 3-month diet vs. before the diet), or an unpaired Student’s *t*-test (comparison between the DD and FD groups); for the variables departing from normality, the Wilcoxon signed-rank test or the Mann–Whitney U test was applied, respectively. The χ^2^ test was used to compare categorical variables.

## 3. Results

### 3.1. Parameter Analysis Between Diabetic Diet (DD) and Fiber-Enriched Diabetic Diet (FD)

The characteristics of the study group are presented in Table 1. The group was predominantly female. The participants were overweight and obese. The DD and FD groups did not differ significantly in age, sex distribution, duration of diabetes, or anthropometric parameters.

#### 3.1.1. Anthropometric Parameters

**Figure 1:**
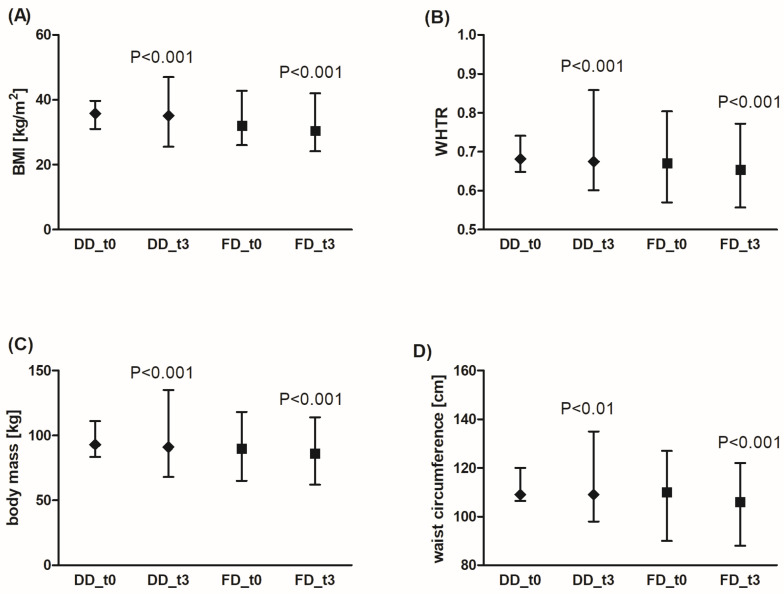
Effects of the diabetic diet (DD) and fiber-enriched diabetic diet (FD) on anthropometric parameters: BMI (**A**), WHTR (**B**), body mass (**C**), and waist circumference (**D**). Both DD and FD significantly decreased the analyzed parameters after 3 months (t3) compared to the time before diets (t0). The data are shown as median and interquartile range. A paired Student’s *t*-test (normal distribution of differences; WHTR, waist circumference for FD) or the Wilcoxon signed-rank test (non-normal distribution of differences; body mass, BMI, waist circumference for DD) was used for statistical analysis. BMI—body mass index; WHTR—waist-to-height ratio.

Adherence to a diabetic diet (DD) under the supervision of a dietitian for 3 months significantly decreased BMI and WHTR (Figure 1A,B), which was linked to a reduction in body mass and waist circumference (Figure 1C,D). Similar results were obtained for the fiber-enriched diabetic diet (FD). There was no significant effect of the diets on WHR. Additionally, FD was more effective in reducing body mass compared to DD (by an average of 2.3% (95% CI: 1.5%; 3.1%) for DD vs. 4.7% (95% CI: 3.5%; 5.9%) for FD, *p* < 0.001), waist circumference (2.0% (95% CI: 1.2%; 3.4%) for DD vs. 3.5% (95% CI: 2.6%; 4.3%) for FD, *p* < 0.01), BMI (2.4% (95% CI: 1.6%; 3.1%) for DD vs. 4.8% (95% CI: 3.6%; 6.0%) for FD, *p* < 0.001), and WHTR (2.0% (95% CI: −1.3%; 0.7%) for DD vs. 3.5% (95% CI: −2.0%; 1.5%) for FD, *p* < 0.01). In summary, although the waist circumference decreased to a greater extent in patients using FD compared to the DD group, this result does not have a significant impact on WHR and WHTR values.

#### 3.1.2. Metabolic Parameters

**Figure 2:**
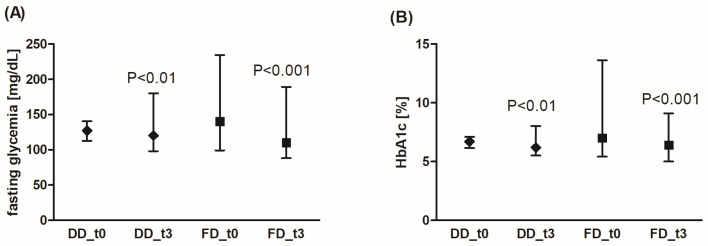
Effects of the diabetic diet (DD) and fiber-enriched diabetic diet (FD) on fasting blood glucose concentration (**A**) and glycated hemoglobin (HbA1c) level (**B**). Both DD and FD significantly decreased the analyzed parameters after 3 months (t3) compared to the time before diet (t0). The data are shown as median and interquartile range. The Wilcoxon signed-rank test was used for statistical analysis.

In both patient groups, using either DD or FD for 3 months, a significant decrease in the fasting glycemia level (Figure 2A) or in HbA1c fraction was observed (Figure 2B). Additionally, FD was more effective in glycemia reduction compared to DD (by an average of 19.9% (95% CI: 14.8%; 25.0%) for FD vs. 5.6% for DD (95% CI: 1.9%; 9.3%), *p* < 0.001). The reduction range of HbA1c level was similar for both diets (by an average of 10.4% (95% CI: 5.3%; 15.5%) for FD vs. 7.4% (95% CI: 2.3%; 12.5%) for DD, not significant).

The analysis of the fasting glucose concentration demonstrated significantly lower values in the DD group compared to the FD group (129 ± 25 mg/dL (MD 127, Q1: Q3 115; 140) vs. 147 ± 30 mg/dL (MD 140, Q1: Q3 128; 157), respectively, *p* = 0.01). Before the diet, fasting glycemia values above 130 mg/dL were observed for 8 patients on DD (32%), and in 17 patients on FD (68%). After 3 months, the glycemia values above 130 mg/dL were found in only eight patients on FD (12%), but in the DD group, the number of patients with such glycemia values did not change significantly (glycemia above 130 mg/dL was still present in seven patients, 28%). The number of patients with HbA1c > 7% decreased in both DD and FD groups by about 50% (7 to 3 for DD, 12 to 6 for FD) after the diet.

#### 3.1.3. Lipid Profile

**Figure 3:**
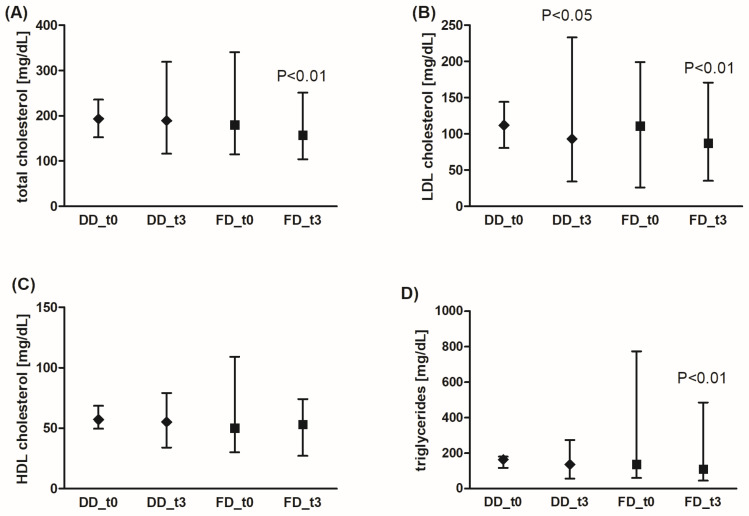
Effects of the diabetic diet (DD) and fiber-enriched diabetic diet (FD) on lipid profile parameters (total (**A**), LDL (**B**), HDL cholesterol (**C**), and triglyceride (**D**) blood concentration). Both DD and FD significantly decreased LDL cholesterol concentration, while only FD significantly reduced total cholesterol and triglyceride concentration after 3 months (t3) compared to the time before diet (t0). The data are shown as median and interquartile range. A paired Student’s *t*-test (normal distribution of differences; total and HDL cholesterol, and triglycerides for DD) or the Wilcoxon signed-rank test (non-normal distribution of differences; total and HDL cholesterol, and triglycerides for FD, LDL cholesterol) was used for statistical analysis.

The concentration of total cholesterol, LDL-cholesterol, and triglycerides was significantly decreased in the FD group, while the standard diabetic diet significantly affected only the LDL-cholesterol level (Figure 3). However, the effects of FD were not statistically significantly better than those of DD. The HDL-cholesterol concentration did not change after the dietary intervention.

#### 3.1.4. Liver Parameters

Liver function in patient groups was monitored by the activity of ALT, AST, and GGTP. Also, FLI and HSI were calculated [16]. HSI is based on ALT, AST, BMI, diabetes, and sex, whereas FLI is based on BMI, waist circumference, GGTP, and triglycerides.

**Figure 4:**
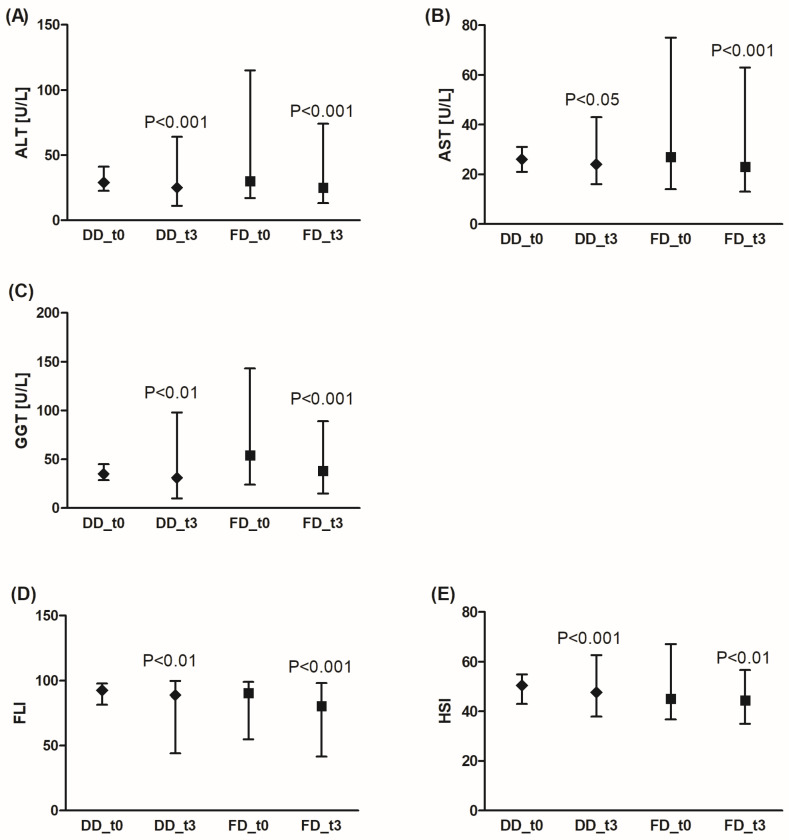
Effects of the diabetic diet (DD) and fiber-enriched diabetic diet (FD) on liver function. Both DD and FD significantly decreased alanine (ALT) (**A**) or aspartate (AST) (**B**) aminotransferase, gamma glutamyl transferase (GGTP) activity (**C**), and FLI (Fatty Liver Index) (**D**) or HSI (Hepatic Steatosis Index) (**E**) after 3 months (t3) compared to before the diet (t0). Data are shown as median and interquartile range. A paired Student’s *t*-test (normal distribution of differences; HSI) or the Wilcoxon signed-rank test (non-normal distribution of differences; ALT, AST, GGT, FLI) was used for statistical analysis.

Both DD and FD significantly improved liver function (Figure 4). A significant reduction was observed for ALT, AST, and GGTP activity. Also, FLI and HSI levels decreased after 3 months of dietary intervention. Additionally, FD was more effective in GGTP activity reduction compared to DD (by an average of 35.7% (95% CI: 28.9%; 42.5%) for FD vs. 1.8% (95% CI: −15.2%; 18.3%) for DD, *p* < 0.001), and FLI decrease was significantly pronounced in the FD group (by an average of 12.6% (95% CI: 7.8%; 17.4%) for FD vs. 4.1% (95% CI: −0.6%; 8.9%) for DD, *p* < 0.001).

The study group consisted predominantly of patients (80%) with ALT, AST, and GGTP activities within the normal range (<56 U/L for AST, <40 U/L for ALT, <60 U/L for GGTP), except for the FD group before the diet (48% patients had GGTP activity above 60 U/L), but after the diet, the number of patients with GGTP activity above 60 U/L decreased to 16%.

#### 3.1.5. Kidney Function

In this study, kidney function was also monitored using estimated glomerular filtration rate (eGFR) value. Neither the FD nor the DD were observed to significantly alter the values of eGFR after 3 months compared to the time before the diets.

### 3.2. Parameter Analysis Between Patients Declaring Adherence to a Diet (Diet Group) and Those Not Following a Diet (Non-Diet Group)

The characteristics of the study group are presented in Table 2. The participants were divided into two subgroups: the Non-Diet group (those claiming not to be on a diet) and the Diet group (those claiming to be on a diet). There were no statistically significant differences between the subgroups with respect to gender, age, place of residence, or education level. However, significant differences were noted for physical activity level, body weight, BMI, waist circumference, and WHTR. It should also be pointed out that the majority of respondents had an improper body weight, while normal body mass was observed in 24% of the Diet group and only 8% of the Non-Diet group. In contrast, excessive body weight was reported in 76% of those claiming to be on a diet and in 92% of those claiming not to be on a diet, including overweight (BMI > 25 kg/m^2^) in 28% vs. 20% and obesity (BMI > 30 kg/m^2^) in 48% vs. 72%, respectively. Among the Diet group, most subjects (76%) reported moderate physical activity, while in the Non-Diet group, most respondents (56%) reported low physical activity (*p* = 0.021). Compared to the Non-Diet group, the Diet group had a statistically significantly lower body weight by 14.47 kg (*p* = 0.016), lower BMI by 4.92 kg/m^2^ (*p* = 0.008), lower waist circumference by 9 cm (*p* = 0.004), and lower WHTR by 0.08 (*p* = 0.001).

#### 3.2.1. Dietary and Lifestyle Habits

In Table 3, the dietary and lifestyle behaviors of the respondents are compared, such as the number and regularity of the meals eaten, snacking between meals, sweetening and salting habits, and alcohol consumption and smoking. Participants in the Diet group more frequently reported eating 4–5 or more meals per day, as well as consuming at least some of them regularly, compared to individuals in the Non-Diet group (60% vs. 36% and 76% vs. 52%, respectively). Interestingly, slightly fewer of those in the Non-Diet group declared snacking between meals at least once a day compared to those in the Diet group (44% vs. 60%). The Diet group, compared to the Non-Diet group, more often decided to refrain from sweetening hot beverages (72% vs. 44%), but more frequently reported salting their food (40% vs. 28%). Subjects following a diet reported slightly more frequent alcohol consumption, but less frequent smoking, compared to those not on a diet (alcohol consumption: 48% vs. 32% and smoking: 24% vs. 36%, respectively). Despite the small differences noted between the groups, they were not statistically significant.

#### 3.2.2. Diet Quality Scores and Nutrition Knowledge

The description of food intake frequency was conducted using the diet indexes characterizing the intensity of food intake frequency of the selected groups of products widely known as healthy, unhealthy, high- and low-GI, and high content of simple sugars and saturated fats [14]. To evaluate the overall diet quality, the pro-Healthy-Diet-Index (pHDI-10) and the non-Healthy-Diet-Index (nHDI-14) scores were established [15], and the high-Glycemic-Diet-Index-7 (hGIDI-7), low-Glycemic-Diet-Index-4 (lGIDI-4), high-Sugar-Diet-Index-4 (hSDI-4), and high-Saturated-Fats-Diet-Index-8 (hSFDI-8) were calculated based on the study by Bykowska-Derda et al. [14]. The indexes were classified on a percentage scale and interpreted in such a way that the higher the value of the index, the higher the intensity of favorable or unfavorable effects on health (Table 4). In all participants and in both study groups, low values were observed for the analyzed dietary indexes, which indicates the weakly marked influence of the protective features of nutrition (pHDI-10, lGIDI-4) as well as the evidence of low contribution of an unhealthy diet (nHDI-10, hGIDI-7, hSFDI-8, and hSDI-4).

The detailed statistical analysis shows that the Diet group had a statistically significant lower intake frequency of products rich in saturated fatty acids compared to the Non-Diet group (12.50% (8.13; 15.38) vs. 18.13% (13.25; 21.25); *p* = 0.015). The Diet group, compared to the Non-Diet group, also had a lower intake frequency of products rich in simple sugars (8.00% (3.13; 21.75) vs. 28.00% (4.25; 38.25)) and high-glycemic-index products (22.89 ± 10.80% vs. 29.48 ± 12.85%). These differences were at the limit of statistical significance, *p* = 0.054 and *p* = 0.055, respectively. The intake frequency of low-glycemic-index products was statistically significantly higher in the Diet group compared to the Non-Diet group (20.50% (14.75; 30.25) vs. 14.00% (8.75; 25.00), *p* = 0.043). In contrast, no statistically significant difference was found between the Diet group and the Non-Diet group regarding the consumption of products considered to be pro-healthy (23.50% (19.70; 30.60) vs. 19.70% (11.20; 24.90); *p* = 0.184). However, among those following a diet, there was a statistically significantly lower intensity of consumption of products considered to be non-healthy compared to the respondents not following a diet (12.58 ± 6.07% vs. 16.72 ± 7.04%; *p* = 0.031).

Moreover, most respondents had a satisfactory level of nutritional knowledge (9–16 points; 80% in the Diet group and 76% in the Non-Diet group), but only 8% of individuals on a diet obtained a good score (17–25 points), while no such scores were recorded among those not following a diet (Table 4). However, the differences in knowledge levels between the groups were not statistically significant.

#### 3.2.3. Health and Nutritional Beliefs

The respondents were also asked to self-assess their health status, dietary habits, and nutrition knowledge (Table 5). Those following a diet were more likely to rate their health as the same or better than that of their peers in the Non-Diet group (72%). In contrast, the majority of participants in the Non-Diet group assessed their health as worse than their peers (64%). Regarding self-assessed nutrition knowledge, the most common response in both groups was “satisfactory” (Non-Diet group: 64%, Diet group: 48%); however, people following a diet were more likely to rate their knowledge as “good” (28% vs. 4%). In both groups, the majority of respondents assessed their dietary habits as good (Diet group: 84%, Non-Diet group: 64%); additionally, individuals in the Diet group were less likely to perceive their dietary habits as bad or very bad compared to those in the Non-Diet group (16% vs. 36%). Although these differences were not statistically significant, they suggest a trend toward a more positive self-assessment of health, greater perceived nutrition knowledge, and higher satisfaction with dietary habits among individuals following a diet.

## 4. Discussion

The available data indicate that there is no single “diet” that can be recommend to patients with T2DM. Furthermore, there are too few high-quality studies comparing appropriate dietary approaches. This is a very important problem from a therapeutic point of view, especially since patients themselves report that following a diet is the most difficult aspect of diabetes treatment [17]. It is worth noting that available guidelines indicate a beneficial effect of a diet with a higher fiber content. Both people with diabetes and those at risk of this disease are encouraged to consume at least 14 g of fiber/1000 kcal, with at least half of the consumed cereals being whole, intact grains [5]. It has been proven that enriching such a diet with an additional portion of fiber (fiber-enriched diet, FD) to the values appropriate for gender and age may be associated with a significant improvement in the control of cardiovascular and metabolic diseases and in the functioning of the gastrointestinal tract [6].

Therefore, we decided to be among the first to compare the effects of the diabetic diet (DD) with the fiber-enriched diabetic diet (FD) on anthropometric, metabolic, liver, and kidney parameters in T2DM patients. We performed the analysis in a group of patients who were under the close control of a dietitian and followed DD or FD, and in patients who were without the close supervision of a dietitian. The results of our study not only confirmed that the use of FD in patients with T2DM is associated with a significant reduction in body weight, BMI, and waist circumference, but also indicated a potential advantage in this respect over the DD. Also, the beneficial effect and advantage of FD over DD in reducing fasting glycemia and similar effectiveness in reduction in glycated hemoglobin HbA1c were observed. The concentration of total cholesterol, LDL cholesterol, and triglycerides was significantly reduced in the FD group, while the standard DD significantly only affected the level of LDL cholesterol. It is worth emphasizing that our observations are consistent with previous studies assessing the use of the FD in patients with T2DM [18,19,20,21]. In addition, it is suggested that reducing the amount of fiber consumed should be considered an important risk factor for the development of NAFLD, as fiber protects the patient not only from the disease itself, but also from its consequences, including hepatocellular carcinoma and premature death [22]. Due to the close relationship between NAFLD and T2DM in terms of etiopathogenesis and the consequences of coexistence [23,24], a beneficial effect of a fiber diet on reducing the risk of NAFLD has been suggested [25]. In our study, we found that FD was more effective in GGTP activity reduction compared to DD, and the lowering of FLI was significantly pronounced in the FD group. Notably, accumulating evidence suggests that dietary fiber may have hepatoprotective properties, reducing hepatic fat accumulation and improving liver function, which are often compromised in T2DM patients [22,26]. Unfortunately, there is still a lack of data assessing the efficacy and safety of other diets in patients with MASLD, especially those with normal body weight [27]. Both FD and DD have not been shown to significantly alter the values of eGFR after 3 months compared to the time before the diet use. It is worth emphasizing that FD and DD are well-established for improving glycemic control, weight, and cardiovascular risk, which over time may slow the progression of renal impairment. However, their immediate impact on eGFR is often limited, due to short observation windows, biological and analytical variability, and the predominance of other renoprotective factors (e.g., antihypertensive therapy). Mechanistically, glycemic control and fiber-driven metabolic improvements exert their renal benefits largely as long-term protection rather than rapid increases in filtration capacity. Therefore, trials and clinical monitoring should emphasize long-term renal outcomes (eGFR trajectory, albuminuria) and integrated cardiometabolic endpoints rather than short-term shifts in eGFR.

Despite these beneficial properties, most traditional dietary guidelines for patients with diabetes have focused primarily on carbohydrate counting and glycemic index regulation, often with limited emphasis on fiber content. While standard diabetes diets offer significant benefits, recent research suggests that supplementing these diets with higher fiber content may further improve metabolic control and liver health. However, the comparative effectiveness of fiber-enriched dietary approaches versus conventional diabetes diets remains understudied in controlled clinical settings. Understanding these differences is crucial to developing personalized nutritional strategies that optimize clinical outcomes and reduce the burden of diabetes-related complications.

In our study, we reported the beneficial effects of FD in acting on a few metabolic parameters; however, there was no difference between DD and FD in lowering Hb levels. Therefore, the question arises whether it is the type of diet or rather the adherence to it that determines its effectiveness. In our study, we showed that T2DM patients declaring the use of a diet (diabetic diet, carbohydrate-restricted diet, sugar-restricted diet, low-glycemic-index diet, and diet that excluded sweets) had lower body mass, BMI, waist circumference, and WHTR, and a higher level of physical activity. We did not find any significant statistical differences between people declaring diet adherence or a lack thereof in terms of dietary behavior patterns, including the number and regularity of meals, snacking between meals, sweetening and salting habits, and alcohol consumption and smoking. People declaring the use of a diet consumed products with a high content of saturated fatty acids, a high content of simple sugars, and a high glycemic index much less often, but it is worth noting that the difference was not statistically significant. Also, the frequency of consuming products with a low glycemic index was significantly higher in the group following a diet, but no such relationship was found for products considered pro-healthy. Additionally, a statistically significantly lower intensity of consumption of products considered to be non-healthy was observed for respondents following a diet. In our study, we found that T2DM patients who were without the close supervision of a dietitian, independently declaring the use of a diet, consumed nutritional products with a weak influence of protective dietary factors as well a low contribution of an unhealthy diet.

Our survey analysis indicated a satisfactory level of nutritional knowledge in this area, but without any difference between the Diet and Non-Diet groups. The analysis of the self-assessment of patients participating in the study indicates moderate awareness of T2DM patients regarding knowledge about nutrition. It is worth emphasizing that these people are largely aware of their health condition and are not satisfied with this fact. For several years, we have been dealing with the problem of following a diet, including in patients with T2DM, and the patients themselves report that following a diet is the most difficult aspect of diabetes treatment. The greatest differences in the perception of patient barriers by providers included poor motivation, frequent consumption of fast food, insufficient family support, and lack of culinary skills—all of which suggest patient inadequacy. However, patients were highly motivated in terms of blood glucose monitoring and desire for nutritional education. Patients identified primary care providers as the primary source of nutritional education, but the providers indicated a lack of time for discussion of diet and preferred other staff to provide education [17].

In our study, we found that diabetics who declared themselves to be not following a diet were characterized by a higher body weight, BMI, waist circumference, and WHTR. However, it is worth noting that over two thirds of people who declared following a diet were overweight, which may indicate limited effectiveness of the nutritional strategies they used. People with diabetes encounter numerous difficulties in maintaining dietary recommendations. Paradoxically, the pressure to comply with dietary recommendations may increase the risk of unreliable reporting of intake. In this group of patients, binge eating, nutritional restraint, and body dissatisfaction are more common [28]. Non-adherence to treatment recommendations is one of the most serious problems in the treatment of people with T2DM. It should be emphasized that lack of trust in the medical team, patients’ misconceptions, and unique experiences and daily challenges of patients prevent full compliance with treatment recommendations [25]. Most often, this is related to the lack of understanding, implementation, and maintenance of the required prerequisites, such as motivation, understanding, health beliefs, self-efficacy, practical goals, and social support [29]. As a result of poor diet, any, even the most advanced, diabetes therapy may prove ineffective. Quite often, patients underestimate the importance of dietary therapy. Many doctors often downplay non-adherence to dietary recommendations, and many of them show great tolerance towards patients who do not follow dietary recommendations. Difficulties in following dietary recommendations are related to issues such as difficult glycemia control due to various factors (physical activity, stress, hormonal changes, comorbidities); the need to change eating habits, especially if the previous ones were inappropriate; eliminating or limiting the consumption of products such as sweets or processed foods; failure to adapt the diet to the individual needs and preferences of the patient; fatigue and routine related to diet control and measuring blood glucose levels; lack of rapid improvement in glycemia control and measuring blood glucose levels, which causes discouragement and weakens motivation; lack of regular education and support from doctors, dietitians, and family in understanding the importance of specific dietary rules; failure to take into account the intellectual abilities to control carbohydrate intake, which is difficult and time-consuming, especially at the beginning; giving up on the diet and choosing unhealthy products in response to difficult emotions (stress, anxiety, frustration); and pressure in social situations, e.g., related to meals in restaurants or during family or social gatherings, when people with diabetes feel pressured to eat what others eat [30].

Current dietary recommendations, also in relation to patients with diabetes, emphasize the importance of not only the quality of consumed products, but also the regularity and variety of meals, the combination of nutrients, and the typical frequency of their consumption [31,32]. The results of our study indicate that people who declare that they follow a diet do not always fully follow these recommendations. Although they more often eat 4–5 or more meals a day, and in a relatively regular manner, they also tend to snack more often between meals, add salt to their meals, and drink alcohol. On the other hand, they sweeten hot beverages less often. It should also be noted that no statistically significant difference was observed between a patient smoking and declaring that they follow a diet, despite the fact that smoking is considered one of the most important risk factors for diabetic complications.

The analysis of the declared dietary habits of the participants in our study, conducted on the basis of the calculated DQS, indicates that patients who followed a diet more often limited their consumption of products perceived as unhealthy, including products with a high content of saturated fatty acids, with a high glycemic index, and containing large amounts of simple sugars. Such dietary choices are consistent with the current recommendations of the world’s diabetes associations [5]. Unfortunately, no significant differences were observed between people who declared that they followed a diet and those who did not, in terms of the frequency of consuming products considered to be pro-health. The results of our study suggest that this may be related to the level of nutritional knowledge of the participants. Importantly, the declaration of following a diet did not always translate into an objective self-assessment of health status, a sense of satisfaction with dietary habits, or satisfactory level of nutritional knowledge. On the other hand, it is worth emphasizing that despite relatively satisfactory nutritional knowledge, and at the same time low self-assessment of health status, some patients with diabetes did not attempt to implement any diet (the group not declaring that they followed a diet). It should be emphasized that following dietary recommendations is associated with numerous health benefits, and supporting and motivating patients to follow them may contribute to extending the period of healthy life [33]. A key role in this process is played by the coherent and active cooperation of the entire team of diabetes care for the patient, including physicians, dietitians, diabetes nurses, and diabetes educators [34,35].

The main limitation of our study was the moderately small number of patients and the predominance of women, which is associated with the limited willingness to use FD by patients with T2DM, mainly men. It should be highlighted that this is consistent with the results of studies indicating that men demonstrate lower health awareness and engagement in preventive behaviors compared to women [36,37]. Furthermore, due to the small size of the study population, the counts for some categorical variables were very low. These values are presented in the tables for illustrative purposes only and were not used for statistical inference. This preliminary study, which demonstrated the important role of regular dietary counseling and monitoring in the use of a low-sugar, high-fiber diet in improving anthropometric and biochemical parameters in T2DM patients, requires further research involving larger groups of patients and longer observation periods.

## 5. Conclusions

Both the standard diabetic diet and the fiber-enriched diet significantly contributed to improving metabolic control in patients with T2DM. Based on the obtained results, it can be assumed that FD is slightly better than DD, especially in terms of improving anthropometric parameters, glycemia and metabolic parameters, and indicators of liver function. Noticeable effects of dietary intervention were observed primarily in the group of patients under the care of a dietitian. Compliance with dietary recommendations remains a fundamental element of T2DM treatment, with an appropriate level of knowledge about proper dietary patterns playing a key role. Although most patients receive basic health education from a doctor as part of their visits to diabetes clinics, only half of them declare that they follow the principles of the diet. Considering the moderate level of nutritional knowledge and low awareness of their health condition in many patients, there is a need to intensify dietary care for people with T2DM.

## Figures and Tables

**Table 1:** Characteristics of T2DM patients before diet intake.

Variables	All Participants (n = 50)	Diabetic Diet (n = 25)	Fiber-Enriched Diabetic Diet (n = 25)	*p*-Value
Age (years)	62.3 ± 8.4	61.8 ± 7.7	62.7 ± 9.1	0.453
Gender:				
Female n (%)	38 (76)	19 (76)	19 (76)	1.000
Male n (%)	12 (24)	6 (24)	6 (24)	
Duration of diabetes (years)	4.5 (2.5; 12.0)	4.0 (2.0; 9.0)	5.0 (2.0; 12.0)	0.714
Waist circumference (cm)	112 ± 10	114 ± 11	110 ± 10	0.676
Body mass (kg)	93.4 ± 15.8	96.7 ± 17.6	90.1 ± 13.2	0.170
BMI (kg/m^2^)	34.6 ± 5.6	36.0 ± 6.1	33.1 ± 4.8	0.232
Overweight (25.0–29.9) n (%)	10 (20)	4 (16)	6 (24)	
Class I obesity (30.0–34.9) n (%)	18 (36)	6 (24)	12 (48)	0.150
Class II obesity (35.0–39.9) n (%)	13 (26)	9 (36)	4 (16)	
Class III obesity (≥40.0) n (%)	9 (18)	6 (24)	3 (12)	
WHR	0.95 ± 0.07	0.94 ± 0.06	0.95 ± 0.08	0.171
WHTR	0.68 ± 0.06	0.70 ± 0.07	0.67 ± 0.06	0.437

Data are presented as mean ± standard deviation or median and interquartile range: MD (Q1; Q3) for quantitative data and as number and percentage for qualitative data. Student’s *t*-test (normal distribution) or the Mann–Whitney U test (non-normal distribution) was used to assess the significance of differences between the diet groups. The χ^2^ test was used for comparisons of categorical variables. BMI—body mass index; WHR—waist-to-hip ratio; WHTR—waist-to-height ratio.

**Table 2:** Basic characteristics of the participants (n = 50).

Variables	All Respondents (n = 50)	Non-Diet Group (n = 25)	Diet Group (n = 25)	*p*-Value
Gender				
Female n (%)	24 (48)	14 (56)	10 (40)	0.258
Male n (%)	26 (52)	11 (44)	15 (60)	
Age (years)	56.8 ± 11.0	55.4 ± 10.0	58.2 ± 12.0	0.374
Place of residence				
Village n (%)	5 (10)	3 (12)	2 (8)	
City 20–100,000 inhabitants n (%)	10 (20)	5 (20)	5 (20)	0.724
City > 100,000 inhabitants n (%)	35 (70)	17 (68)	18 (72)	
Education				
Primary n (%)	5 (10)	4 (16)	1 (4)	
Vocational n (%)	9 (18)	5 (20)	4 (16)	0.247
Secondary n (%)	18 (36)	10 (40)	8 (32)	
Higher n (%)	18 (36)	6 (24)	12 (48)	
Physical activity				
Low n (%)	20 (40)	14 (56)	6 (24)	
Moderate n (%)	30 (60)	11 (44)	19 (76)	0.021
High n (%)	0 (-)	0 (-)	0 (-)	
Body mass (kg)	93.24 ± 21.41	100.63 ± 22.18	86.16 ± 18.39	0.016
BMI (kg/m^2^)	31.88 ± 6.56	34.34 ± 6.83	29.42 ± 5.37	0.008
Normal range (18.5–24.9) n (%)	8 (16)	2 (8)	6 (24)	
Overweight (25.0–29.9) n (%)	12 (24)	5 (20)	7 (28)	
Class I obesity (30.0–34.9) n (%)	18 (36)	10 (40)	8 (32)	
Class II obesity (35.0–39.9) n (%)	6 (12)	2 (8)	4 (16)	
Class III obesity (≥40.0) n (%)	6 (12)	6 (24)	0 (-)	
Waist circumference (cm)	108.0 (100.0; 116.0)	110.0 (107.0; 120.0)	101.0 (90.0; 110.0)	0.004
WHTR	0.63 ± 0.08	0.67 ± 0.08	0.59 ± 0.06	0.001
Moderate health risk (<0.5) n (%)	4 (8)	1 (4)	3 (12)	
Increased health risk (≥0.5) n (%)	46 (92)	24 (96)	22 (88)	

Data are presented as mean and standard deviation (M ± SD; normal distribution) or median and interquartile range (MD (Q1; Q3); non-normal distribution) for quantitative data and as number and percentage for qualitative data. Student’s *t*-test (normal distribution) or the Mann–Whitney U test (non-normal distribution) for numerical variables and the χ^2^ test for categorical variables were used to assess the significance of differences between two the groups (Non-Diet group vs. Diet group). BMI—body mass index; WHTR—waist-to-height ratio.

**Table 3:** Comparison of dietary and lifestyle habits in the Non-Diet group (n = 25) and the Diet group (n = 25).

Dietary and Lifestyle Habits	All Respondents (n = 50)	Non-Diet Group (n = 25)	Diet Group (n = 25)	*p*-Value
Number of meals consumed per day				
2 meals n (%)	5 (10)	3 (12)	2 (8)	
3 meals n (%)	21 (42)	13 (52)	8 (32)	0.363
4 meals n (%)	20 (40)	8 (32)	12 (48)	
5 meals or more n (%)	4 (8)	1 (4)	3 (12)	
Eating meals at regular times				
No n (%)	18 (36)	12 (48)	6 (24)	0.209
Yes, but only some of them n (%)	22 (44)	9 (36)	13 (52)	
Yes, all of them n (%)	10 (20)	4 (16)	6 (24)	
Snacking between meals				
Never n (%)	1 (2)	1 (4)	0 (-)	
1–3 times per month n (%)	4 (8)	3 (12)	1 (4)	
Once per week n (%)	7 (14)	4 (16)	3 (12)	0.699
Several times per week n (%)	12 (24)	6 (24)	6 (24)	
Once per day n (%)	20 (40)	9 (36)	11 (44)	
Several times per day n (%)	6 (12)	2 (8)	4 (16)	
Sweetening hot beverages				
No n (%)	29 (58)	11 (44)	18 (72)	
Yes, one teaspoon of sugar or honey n (%)	14 (28)	9 (36)	5 (20)	0.120
Yes, two or more teaspoons of sugar or honey n (%)	3 (6)	3 (12)	0 (-)	
Yes, sweeteners n (%)	4 (8)	2 (8)	2 (8)	
Salting prepared meals				
No n (%)	33 (66)	18 (72)	15 (60)	0.321
Yes, but only sometimes n (%)	16 (32)	6 (24)	10 (40)	
Yes, most meals n (%)	1 (2)	1 (4)	0 (-)	
Alcohol consumption				
No n (%)	30 (60)	17 (68)	13 (52)	0.248
Yes n (%)	20 (40)	8 (32)	12 (48)	
Smoking				
No n (%)	35 (70)	16 (64)	19 (76)	0.470
Yes n (%)	15 (30)	9 (36)	6 (24)	

Data are presented as number and percentage for qualitative data. The χ^2^ test was used to assess the significance of differences between two groups (Non-Diet group vs. Diet group).

**Table 4:** Comparison of the nutrition knowledge level and values of diet quality scores (pHDI-10, nHDI-14, hGIDI-7, lGIDI-4, hSDI-4, hSFDI-8) in the Non-Diet group and the Diet group.

Diet Quality Scores and Nutrition Knowledge	All Respondents (n = 50)	Non-Diet Group (n = 25)	Diet Group (n = 25)	*p*-Value
pHDI-10 (% points)	21.50 (13.50; 28.20)	19.70 (11.20; 24.90)	23.50 (19.70; 30.60)	0.184
Low (0–33%) n (%)	43 (86)	21 (84)	22 (88)	
Moderate (34–66%) n (%)	7 (14)	4 (16)	3 (12)	
High (67–100%) n (%)	0 (-)	0 (-)	0 (-)	
nHDI-14 (% points)	14.65 ± 6.83	16.72 ± 7.04	12.58 ± 6.07	0.031
Low (0–33%) n (%)	49 (98)	24 (96)	25 (100)	
Moderate (34–66%) n (%)	1 (2)	1 (4)	0 (-)	
High (67–100%) n (%)	0 (-)	0 (-)	0 (-)	
hGIDI-7 (% points)	26.19 ± 12.21	29.48 ± 12.85	22.89 ± 10.80	0.055
Low (0–33%) n (%)	39 (78)	17 (68)	22 (88)	
Moderate (34–66%) n (%)	11 (22)	8 (32)	3 (12)	
High (67–100%) n (%)	0 (-)	0 (-)	0 (-)	
lGIDI-4 (% points)	19.50 (14.00; 27.50)	14.00 (8.75; 25.00)	20.50 (14.75; 30.25)	0.043
Low (0–33%) n (%)	43 (86)	23 (92)	20 (80)	
Moderate (34–66%) n (%)	6 (12)	2 (8)	4 (16)	
High (67–100%) n (%)	1 (2)	0 (-)	1 (4)	
hSDI-4 (% points)	14.50 (3.13; 30.63)	28.00 (4.25; 38.25)	8.00 (3.13; 21.75)	0.054
Low (0–33%) n (%)	41 (82)	16 (64)	25 (100)	
Moderate (34–66%) n (%)	9 (18)	9 (36)	0 (-)	
High (67–100%) n (%)	0 (-)	0 (-)	0 (-)	
hSFDI-8 (% points)	13.94 (8.63; 19.88)	18.13 (13.25; 21.25)	12.50 (8.13; 15.38)	0.015
Low (0–33%) n (%)	48 (96)	24 (96)	24 (96)	
Moderate (34–66%) n (%)	2 (4)	1 (4)	1 (4)	
High (67–100%) n (%)	0 (-)	0 (-)	0 (-)	
Nutrition knowledge level (points)	11.08 ± 3.35	10.84 ± 3.45	11.32 ± 3.30	0.617
Unsatisfactory (0–8) n (%)	9 (18)	6 (24)	3 (12)	
Satisfactory (9–16) n (%)	39 (78)	19 (76)	20 (80)	
Good (17–25) n (%)	2 (4)	0 (-)	2 (8)	

Data are presented as mean and standard deviation (M ± SD; normal distribution) or median and interquartile range (MD (Q1; Q3); non-normal distribution) for quantitative data and as number and percentage for qualitative data. Student’s *t*-test (normal distribution) or the Mann–Whitney U test (non-normal distribution) was used to assess the significance of differences between the two groups (Non-Diet group vs. Diet group). pHDI-10—pro-Healthy-Diet-Index-10; nHDI-14—non-Healthy-Diet-Index-14; hGIDI-7—high-Glycemic-Diet-Index-7; lGIDI-4—low-Glycemic-Diet-Index-4; hSDI-4—high-Sugar-Diet-Index-4; hSFDI-8—high-Saturated-Fats-Diet-Index-8.

**Table 5:** Comparison of self-reported health status compared to peers, nutrition knowledge, and nutrition in the Non-Diet group (n = 25) and the Diet group (n = 25).

Health and Nutritional Beliefs	All Respondents (n = 50)	Non-Diet Group (n = 25)	Diet Group (n = 25)	*p*-Value
Self-assessment of health status compared to peers				
Worse than peers n (%)	23 (46)	16 (64)	7 (28)	
Same as peers n (%)	24 (48)	8 (32)	16 (64)	0.072
Better than peers n (%)	3 (6)	1 (4)	2 (8)	
Self-assessment of nutrition knowledge				
Unsatisfactory n (%)	13 (26)	7 (28)	6 (24)	
Satisfactory n (%)	28 (56)	16 (64)	12 (48)	0.105
Good n (%)	8 (16)	1 (4)	7 (28)	
Very good n (%)	1 (2)	1 (4)	0 (-)	
Self-assessment of nutrition				
Very bad n (%)	1 (2)	1 (4)	0 (-)	
Bad n (%)	12 (24)	8 (32)	4 (16)	0.222
Good n (%)	37 (74)	16 (64)	21 (84)	
Very good n (%)	0 (-)	0 (-)	0 (-)	

Data are presented as number and percentage for qualitative data. The χ^2^ test was used to assess the significance of differences between the two groups (Non-Diet group vs. Diet group).

## Data Availability

The original contributions presented in this study are included in the article/Supplementary Material. Further inquiries can be directed to the corresponding author.

## Supplementary Materials

The following supporting information can be downloaded at https://www.mdpi.com/article/10.3390/nu18020222/s1. Table S1: Comorbidities and medications used in the T2DM patient group.

## Abbreviations

The following abbreviations are used in this manuscript:

ALT	alanine aminotransferase
AST	aspartate aminotransferase
BMI	body mass index
DD	diabetic diet
DQS	diet quality scores
eGFR	glomerular filtration rate
FD	fiber-enriched diabetic diet
FLI	Fatty Liver Index
GGTP	gamma glutamyl transferase
HbA1c	glycated hemoglobin
hGIDI-7	high-Glycemic-Diet-Index-7
hSDI-4	high-Sugar-Diet-Index-4
hSFDI-8	high-Saturated-Fats-Diet-Index-8
HIS	Hepatic Steatosis Index
lGIDI-4	low-Glycemic-Diet-Index-4
NAFLD	non-alcoholic fatty liver disease
nHDI-14	non-Healthy-Diet-Index
pHDI-10	pro-Healthy-Diet-Index
T2DM	type 2 diabetes mellitus
WHTR	waist-to-height ratio
