# Supplementary material for: Good Dietary Control Significantly Improves Anthropometric and Metabolic Parameters and Liver Function in Patients with Type 2 Diabetes Mellitus—A Pilot Study
Source: Nutrients. 2026 Jan 10;18(2):222. doi: 10.3390/nu18020222 (PMC12845430; doi:10.3390/nu18020222)
Supplement: Supplementary file 1 [file nutrients-18-00222-s001.zip › nutrients-4000658-supplementary.pdf]

Table S1. Comorbidities and medications used in the T2DM patient group.

Variables	Diabetic diet (n = 25)	Fiber-enriched diabetic diet (n = 25)	P-value
Dyslipidemia n (%)	10 (40)	12 (48)	0.776
NAFLD	16 (64)	15 (60)	1.000
Medications:			
Metformin n (%)	20 (80)	20 (80)	1.000
PSU n (%)	13 (52)	9 (36)	0.393
Insulin n (%)	2 (8)	2 (28)	0.138
DPP4 inhibitors n (%)	2 (8)	3 (12)	1.000
Statins n (%)	7 (28)	10 (40)	0.551
Fibrates n (%)	2 (8)	4 (16)	0.667
SGLT2 inhibitors n (%)	4 (16)	4 (16)	1.000

Data are presented as numbers and percentages. Fisher's exact test was used for comparisons of categorical variables. NAFLD–non-alcoholic fatty liver disease.
